# On the Dental Movement in England

**Published:** 1858-11

**Authors:** 


### ON THE DENTAL MOVEMENT IN ENGLAND.

To the Editors of the Dental News Letter.

*Gentlemen:*—In your April number I observe a very heart-stirring suggestion, to assist in which, I should think our brethren on this side of the Atlantic would enthusiastically unite. Twelve months ago, I can say for certain, the College of Dentists would have hailed a visit from the dentists of America with immense pleasure, and I doubt not would still do so. *But* (I hate the horrid little word) the poor College is sadly shorn of its greatness. Jealousy and illiberality without, treachery and imbecility within, have reduced that noble and liberal institution which promised, and would have fulfilled its promise, of great and beneficial results, to a *wreck* of its former self.

With your liberal trans-atlantic notions it may seem paradoxical that an institution, commenced by a public meeting of the profession, should have met with so much animus, even to those here not immediately concerned in the movement, this is difficult to conceive; but to the initiated it is forcibly perspicuous. And, that no misunderstanding may exist in America, whose noble institutions we fain would have emulated, I shall briefly give an outline of the movement—its rise and fall.

I believe it is admitted on all sides, that for many years the want of status and union has been both felt and acknowledged; but how to bring about this desirable concord was the great difficulty; there being certain individuals who seemed to imagine they held a prescriptive or vested right to the dental loaves and fishes. "But to our Tale."

In the *Lancet*, August 25th, 1855, a letter appeared from Samuel Lee Rymer, entitled "The necessity for a College of Dental Surgery." This was followed upon the 8th of September by a letter from myself, on "Dental Diplomas." But those letters did little to agitate the matter, and it is probable that, had not Mr. Rymer, at his own expense, called a public meeting upon the 22d of September, at the Free Mason's Tavern, things would have remained in statu quo. I was present at this rather numerously-attended meeting, at which there was but a sprinkling of the leading practitioners. However, business proceeded; a committee was formed to organize the movement; and, in compliment to the gentlemen in high positions, although absent, most of them were provisionally nominated upon the committee. During the proceedings, a young gentleman, seemingly not known to those present, remarked, that he believed a few gentlemen of the profession had taken upon themselves to petition the College of Surgeons to grant a special diploma in dental surgery, as in midwifery. This secret proceeding on the part of those gentlemen did not seem to meet the approval of the assembly. The appointments were nevertheless concluded, placing certain power in the hands of this publicly appointed committee; to which I was elected Chairman, and Mr. Rymer, Secretary. The work now proceeded merrily; progressing fast under the appellation of the "Institute of Dental Science." Hundreds of dentists from all parts sent in their adhesion; until, like unto a snow-ball rolled on, its dimensions grew gigantic; we being then supported in our labors by the *British Journal of Dental Science*, and opposed on every available point by the twelve or eighteen gentlemen who had petitioned the College of Surgeons. These gentlemen speedily set about forming that which is now called the "Odontological Society," having for its object, the avowed endeavor to crush the rising college in the bud, by the weight of its opposing influences. But this was not to be so easily effected; the work still proceeded cheerily.

A public meeting was again called upon the 11th of November, for the purpose of forming the association, or rather christening the one, at that time, pretty well formed. Mr. James Robinson was called to the chair. The rules were presented to the meeting, discussed and returned to the executive, with the suggested alterations, to be re-presented in form to the profession at a future meeting. During this interval, it must be noticed that the *British Journal of Dental Science* was *purchased* by the promoters of the rival society; so that now, instead of its support, we had to endure its vituperation. Notwithstanding these drawbacks, our open, fair and liberal proceedings carried with them to the profession conviction of propriety, and we kept rapidly on the increase. From letters and arguments, it was decided, that as a society had started into existence, and a dental educational establishment was much needed, our institute should become a College. "But, Sirs, this pleased them was't ava," and a dire animosity set in upon us.

On the 16th of December, 1856, another public meeting was held, called by the provisional committee, that they might render account of their stewardship—myself, as Chairman, presiding. The rules were now passed, and the college formed; about two hundred members were enrolled; Mr. James Robinson elected President; and almost an entirely new council nominated. The members of the provisional committee, with few exceptions, were overlooked, in order to make room for that element which proved its ultimate destruction.

On February the 14th, the inaugural meeting took place, when the President made a very appropriate address. Alas! for poor human nature, how little can we foretell what a day may bring forth.

For about ten months, the college continued to progress, and bid fair to realize all our fondest hopes and expectations; but there was an adder in its path—a viper in its boscm. Measures began to eminate from, and new laws to be enacted by, the council, which were distasteful to the members. An educational curriculum was proposed, which required many more qualifications than would even be necessary to pass Physicians' Hall. Indeed, a course of study, that seemed to have the effect of thoroughly frightening its concocters into a compromise with the "Odontological Society," unless it might have been a part of a deeply laid plot to sell the college to that body. Be that as it may, a little odontological cajolery and a good dinner completely bought over the President to the amalgamation question, which, in plain English, amounts to this: "If you will agree to sink the college, which seems destined to carry so much weight, and allow it to become a mere society, we will extend the hand of patronage to such of you as we think may deserve our notice; by which means, the great will remain great, the little small, and we shall no longer be tormented with the fear that rising talent will be brought forward, and our positions and prestige threatened."

In January, 1858, the Odontological Dinner took place, to which were invited Mr. Robinson and a few of his friends and privy council. They were feasted and flattered. This sealed the fate of the College. On account of the extreme unfairness of the propositions, and the arbitrary method taken to force them, the amalgamation was rejected by the members. The President resigned, taking with him nearly all the influential portion of the council, who, no doubt, had been well tutored into exclusiveness by the destructives; driving the remaining councilmen into the highways and by-ways to recruit their ranks. Thus, and for the present, is the moral prestige of the college gone. Still, its friends thinks "there is life in the old dog yet," in spite of its dreadful fall. Let us hope so, and that, when resuscitated, the hands that threw it over the cliff, may be painted in red upon its altar, as a memento to all those who may hereafter reap benefit from its instructions.

And now, gentlemen, I leave you to judge whether a visit would be well-timed, in the present disjointed state of the profession in England. Time works wonders; let us hope things will mend. The contemplated World's Exhibition of '61 may prove an additional inducement and a fitter time for our brethren of the West to visit us.

I remain, gentlemen, most cordially yours,

DONALDSON MACKENZIE.

Saville Row, London, July 9th, 1858.

---

From the above it would seem that the dental educational movement in England has met with serious checks and that the desire to connect both organizations, and thus concentrate effort, has led some (whose earnestness in the cause of dental progress we can not doubt) into unreasonable concessions, or to the acceptance of what is considered by many as humiliating conditions, in the furtherance of this very desirable object. We trust, however, that these checks will prove but temporary, and that the college will soon be reinstated in professional favor and support, as, indeed, our correspondent gives us reason to hope.

In reference to our suggestion of a meeting of the American Dental Convention in London, it will be remembered that we named 1859 or 1860 as the time, but as a year is of no great importance in such connection, and the hearty coöperation of the British dentists all important, we readily accept the suggestion made by our correspondent as above, and submit that August, 1861, be fixed upon as the best time for the meeting of the "World's Dental Convention" in London.—*News Letter.*

